# *CLCA1* mediates the regulatory effect of IL-13 on pediatric asthma

**DOI:** 10.3389/fped.2022.959439

**Published:** 2022-10-12

**Authors:** Yanan Xu, Lili Cao, Jiong Chen, Danyan Jiang, Peisen Ruan, Qinsong Ye

**Affiliations:** ^1^ Department of Research, Ningbo Women's and Children's Hospital, Ningbo, China; ^2^ Department of PICU, Ningbo Women's and Children's Hospital, Ningbo, China; ^3^ Department of Pediatrics 3, Ningbo Women's and Children's Hospital, Ningbo, China; ^4^ Department of Asthma Center, Ningbo Women's and Children's Hospital, Ningbo, China

**Keywords:** *CLCA1*, pediatric asthma, IL-13, IL-4, *in vitro*

## Abstract

**Objective:**

*CLCA1* is a secreted protein with protease activity, and its expression is associated with inflammatory airway diseases. This study aimed to investigate the role of *CLCA1* and IL-13 in pediatric asthma.

**Methods:**

In asthmatic and healthy children, the correlation between *CLCA1* expression and blood IL-4, and IL-13 levels were investigated by serological analyses such as RT-qPCR and ELISA. The effects on the activity and apoptosis of bronchial epithelial cells following IL-13 stimulation were explored *in vitro* by the CCK-8 assay and flow cytometry, respectively. *CLCA1* siRNA was used to knock down the expression level of bronchial epithelial cells and the effect of IL-13 stimulation on these cells was assessed by the CCK-8 assay and flow cytometry.

**Results:**

*CLCA1*, IL-4, and IL-13 were highly expressed in the serum of children with asthma. *CLCA1* expression was highly correlated to serum IL-13. IL-13 stimulation reduced the activity of bronchial epithelial cells *in vitro* and promoted apoptosis. Lastly, knockdown of *CLCA1* rescued the IL-13-induced decrease in activity and apoptosis.

**Conclusion:**

*CLCA1* is highly expressed in children with asthma and mediates the contributory effect of IL-13 on the occurrence and development of pediatric asthma.

## Introduction

Worldwide, pediatric asthma is the most common childhood chronic lower respiratory disease (1). Pediatric asthma begins early in life and may progress or resolve over time. Approximately, 50% of children with asthma become asymptomatic over time. However, some asthma symptoms may persist throughout the life of the child. In atopic and severe cases, asthma has a disproportionate impact on a patient's quality of life (2). Pediatric asthma differs from adult asthma due to the lack of mature respiratory and immune systems in children, lack of good evidence, and difficulty in establishing a diagnosis to provide medication (3). However, genetic differences are hypothesized to play a role in the onset and progression of pediatric asthma.

Calcium-activated chloride channel regulator (CLCA) proteins are a family of secreted self-cleaving and zinc-dependent metalloproteases that activate calcium-dependent chloride currents in mammalian cells (4, 5). *CLCA1* is the most studied member of the CLCA family and mainly plays an important role in human respiratory diseases. Furthermore, previous studies have shown that *CLCA1* is an important secretory mediator in asthma, chronic obstructive pulmonary disease, cystic fibrosis, and other diseases that exhibit increased mucus production (6, 7). In addition to regulating airway mucus secretion, *CLCA1* may also be involved in the regulation of tissue inflammation during the innate immune response through the regulation of cytokine and chemokine production (8). The secreted form of *CLCA1* acts as a signaling ligand that activates U-937 cells, a human monocytic cell line, as well as primary cultured porcine alveolar macrophages in a dose-dependent manner and increases proinflammatory interleukin (IL)-1β, IL-6, IL- 8, and tumor necrosis factor-α (TNF-α) expression, which act as pleiotropic factors in lung inflammation [24349445]. Therefore, *CLCA1* is considered to have an important role in respiratory diseases; however, its role in pediatric asthma has not been elucidated.

Additionally, type 2 asthma, the most common form of asthma, is characterized by airway and blood eosinophilia, of which the type 2 cytokine, IL-13, is the main biomarker (9). In childhood, type 2 asthma is usually caused by allergic sensitization and exposure to inhaled allergens. This sensitization is driven by allergen-specific CD4^+^ Th2 cells and is evidenced by the presence of allergen-specific IgE in serum. Additionally, multiple studies have reported evidence to show that Th2 cell-related cytokines produced by CD4^+^ T cells are associated with asthma (10–12). In Guangdong, Wu et al. reported that the IL-13 promoter polymorphisms are important contributors to childhood asthma. Furthermore, the authors showed that the mutant T allele is associated with asthma and can increase serum IgE levels (13). Additionally, in Xinjiang Uygur, several studies have found that IL-4 and IL-13 gene polymorphisms are associated with childhood asthma (14). The IL-13 gene is located on human chromosome 5q23-q31 and consists of 4 exons and 3 introns, encoding a 132 amino acid protein (15). IL-13 is adjacent to the IL-4, IL-3, IL-5, IL-9, and GM-CSF genes, forming a cytokine gene cluster. As stated above, IL-13 is mainly secreted by activated CD4^+^ T cells (Th2), and its biological function is to inhibit the release of inflammatory cytokines and chemical factors from monocytes, induce B cell proliferation and differentiation, promote IgE synthesis and the production of several adhesion molecules that are expressed in endothelial cells (16).

In this paper, we investigated the expression levels of *CLCA1*, IL-4, and IL-13 in pediatric asthma. We found that the expression of *CLCA1* and IL-13 were positively correlated in pediatric asthma relative to healthy controls. At the same time, *in vitro* IL-13 stimulation reduced the activity of bronchial epithelial cells and promoted apoptosis. Knockdown of *CLCA1* alleviated the IL-13-induced decrease in activity and apoptosis of bronchial epithelial cells. Together, these results demonstrate that *CLCA1* may be a potential therapeutic target in pediatric asthma.

## Materials and methods

### Clinical samples

In this study, 34 patients (age 2–7 years old) diagnosed with asthma were selected from individuals with pediatric asthma (Ningbo Women's and Children's Hospital). All patients were examined and diagnosed as asthma by clinicians according to the clinical guidelines of the Global Initiative for Asthma (GINA, 2019). In addition, 35 individuals with an age range of 2–7 years (matched with patients) were selected as normal controls. The individuals in the normal control group showed no signs or symptoms of asthma. As shown in Supplementary materials.This study was approved by the hospital ethics committee and all patients signed informed consent.

### Genetic screening

The GSE103166 dataset was retrieved from the GEO database (https://www.ncbi.nlm.nih.gov/geo/). Differentially expressed mRNA transcripts were identified using the limma package deployed in R (version: 3.40.2). “Adjusted *p * < 0.05 and log2 (fold change) > 1 or log2(fold change) < −1” was defined as a threshold mRNA differential expression screen.

### Cell culture and transfection

Bronchial epithelial cell lines (BEAS-2B cells) were purchased from the American Type Culture Collection (ATCC; Manassas, VA, USA). The cells were cultured in RPMI-1640 medium (Hyclone, USA) supplemented with 10% fetal bovine serum (Gibco, USA). The cells were cultured in a humidified atmosphere at 37 °C supplemented with 5% CO2. For *in vitro* stimulation experiments, the cells were treated with recombinant IL-13 (2 ng/ml, Thermo Fisher Scientific, USA). CLCA1 siRNA (Tsingke, China) and control RNA were transfected into BEAS-2B cells in logarithmic growth phase.Transfections were performed using Lipofectamine 3,000 transfection reagent (Invitrogen, USA) according to the manufacturer's protocol.

### ELISA

Peripheral venous blood and upper serum were taken, and the serum IL-4 and IL-13 levels of the subjects were detected by ELISA in strict accordance with the instructions of the ELISA kit (Lianke, China), and the absorbance value at 450 nm wavelength was measured by a microplate reader. Results The standard curve was drawn to calculate the levels of IL-4 and IL-13.

### qRT-PCR

Total RNA was extracted from the blood and cells using the TRIzol reagent (Invitrogen, USA) following the manufacturer's protocol. The concentration and purity of RNA were detected by a trace nucleic acid protein analyzer. To reverse transcribe the RNA, 500 ng RNA was used to synthesize cDNA using the TaKaRa Reverse Transcription Kit (Takara, Japan). For qRT-PCR, the reaction was carried out according to the instructions of the TaKaRa fluorescence quantitative PCR detection kit (Takara, Japan) using the newly synthesized cDNA as a template. The experiment was repeated three times and GAPDH was used as an internal reference. The forward primer sequence for CLCA1 was CGTCAAATACTCCCCATCGT (5′ to 3′) and the reverse primer sequence was GCTGATGTTCTGGTTGCTGA (5′ to 3′). The forward primer sequence for GAPDH was ACCCACTCCTCCACCTTTGAC (5′ to 3′) and the reverse primer sequence was TGTTGCTGTAGCCAAATTCGTT (5′ to 3′). The relative expression levels of CLCA1 were assessed using the 2-ΔΔCt method.

### CCK-8 assay

BEAS-2B cells in the logarithmic growth phase were diluted and spread at a volume of 200 *μ*l per well in a 96-well plate. Each group consisted of 3 wells. At approximately 70% confluency, the cells were transfected with siRNA was added. After culturing for 0, 24, 48, 72, and 48 h, 10 *μ*l of CCK-8 solution (Dojindo, Japan) was added to each well, and the cells were incubated at 37 °C for 2 h in the dark. Thereafter, absorbance was measured at 450 nm in a microplate reader (Labsystems, Finland).

### Statistical analysis

Statistical analysis was performed using GraphPad Prism 6 software. Data are presented as mean ± SD and all experiments were performed in triplicate. The chi-square test was used to assess the relationship between normal children and clinical characteristics of children with asthma. Differences between the two groups were analyzed using Student's *t*-test. *p* < 0.05 or *p* < 0.01 indicated statistical significance.

## Results

### Demographic information

The clinical characteristics of patients and normal controls are summarized in Table 1. Hypersensitivity C-reactive protein (*p* < 0.0209), Immunoglobulin IgE (*p *< 0.0003), Hemoglobin (*p* < 0.0001), White blood cell count (*p* < 0.0001), Neutrophil percentage (*p* < 0.0001) and Lymphocytes percentage (*p* < 0.0001) levels were significantly increased.

**Table 1:** Clinical characteristics.

	Controls (*n* = 35)	Asthmatic children (*n* = 34)	*p* value (Student *t* test)
Age	4.41 ± 1.02	4.61 ± 1.12	–
Sex (M/F)	20/15	20/14	–
Hypersensitivity C-reactive protein (mg/L)	3.84 ± 3.88	7.33 ± 7.76	0.0209
Immunoglobulin IgE (IU/ml)	54.15 ± 49.36	322.5 ± 403.66	0.0003
Percantage of eosinophils	2.07 ± 2.02	2.47 ± 1.93	0.4389
Percantage of basophils	0.25 ± 0.44	0.36 ± 0.49	0.433
Hemoglobin (g/dl)	12 ± 1.15	13.06 ± 0.86	0.0001
White bood cell count	5.36 ± 1.83	11.49 ± 5.25	<0.0001
Platelet count	285.08 ± 114.05	287.39 ± 92.19	0.9599
Neutrophil percentage	38.06 ± 14.50	60.35 ± 14.16	<0.0001
Lymphocytes percentage	52.54 ± 13.17	30.72 ± 14.17	<0.0001
Asthma severity	–	3/19/12	–

### Profile of mRNA in the airway epithelium of asthma patients and normal people

**Figure 1:**
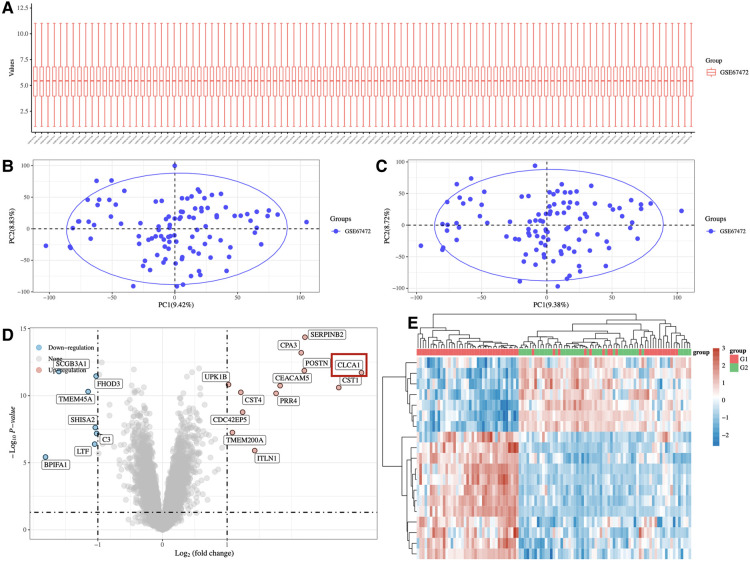
Profile of mRNA in the airway epithelium of asthma patients and normal people. (**A**) Heatmap depicting differentially expressed mRNAs in the airway epithelial tissues of 65 asthma patients and 43 normal airway epithelial tissues. (**B**) Volcano plot depicting the fold change values and *p*-values of differentially expressed genes.

To identify differentially expressed genes in asthmatic patients vs. healthy control, pediatric asthma mRNA microarray data (Accession No. GSE67472) was extracted from the GEO database. Bioinformatics analysis was to annotate, normalize and analyze the differential expression of the data. The generated cluster heatmaps indicated that many mRNAs were differentially expressed in the airway epithelial tissue of asthmatic vs. healthy controls (Figure 1A). Additionally, the generated volcano plots highlighted significant differentially expressed genes between the asthmatic and healthy control groups. Of these significantly differentially expressed genes, 12 were upregulated and 7 were downregulated. *CLCA1* exhibited the greatest fold difference between the asthma children and healthy controls and was therefore selected as the candidate gene for our study (Figure 1B).

### *CLCA1* is highly expressed in the serum of children with asthma

**Figure 2:**
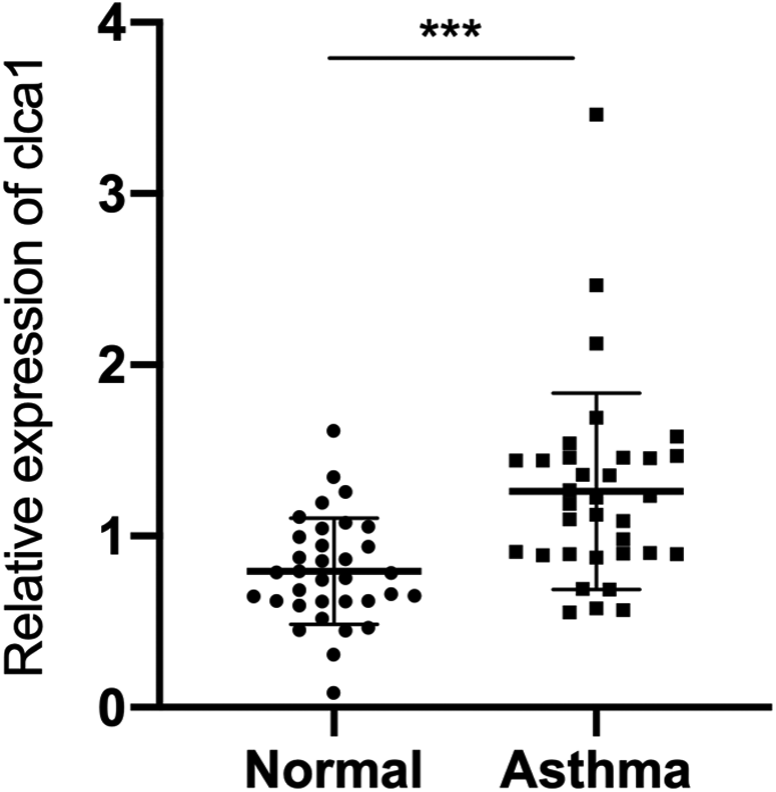
*CLCA1* is highly expressed in the serum of children with asthma. RT-qPCR analysis of *CLCA1* expression in airway epithelial tissues of 34 pairs of healthy children and children with asthma. ***p < 0.001.

Bioinformatic analysis showed that *CLCA1* is highly expressed in asthmatic children. In agreement with these data, qRT-PCR analysis healthy children and children with asthma confirmed that *CLCA1* is significantly expressed in the blood of children with asthma (Figure 2).

### IL-4 and IL-13 are highly expressed in the serum of children with asthma

**Figure 3:**
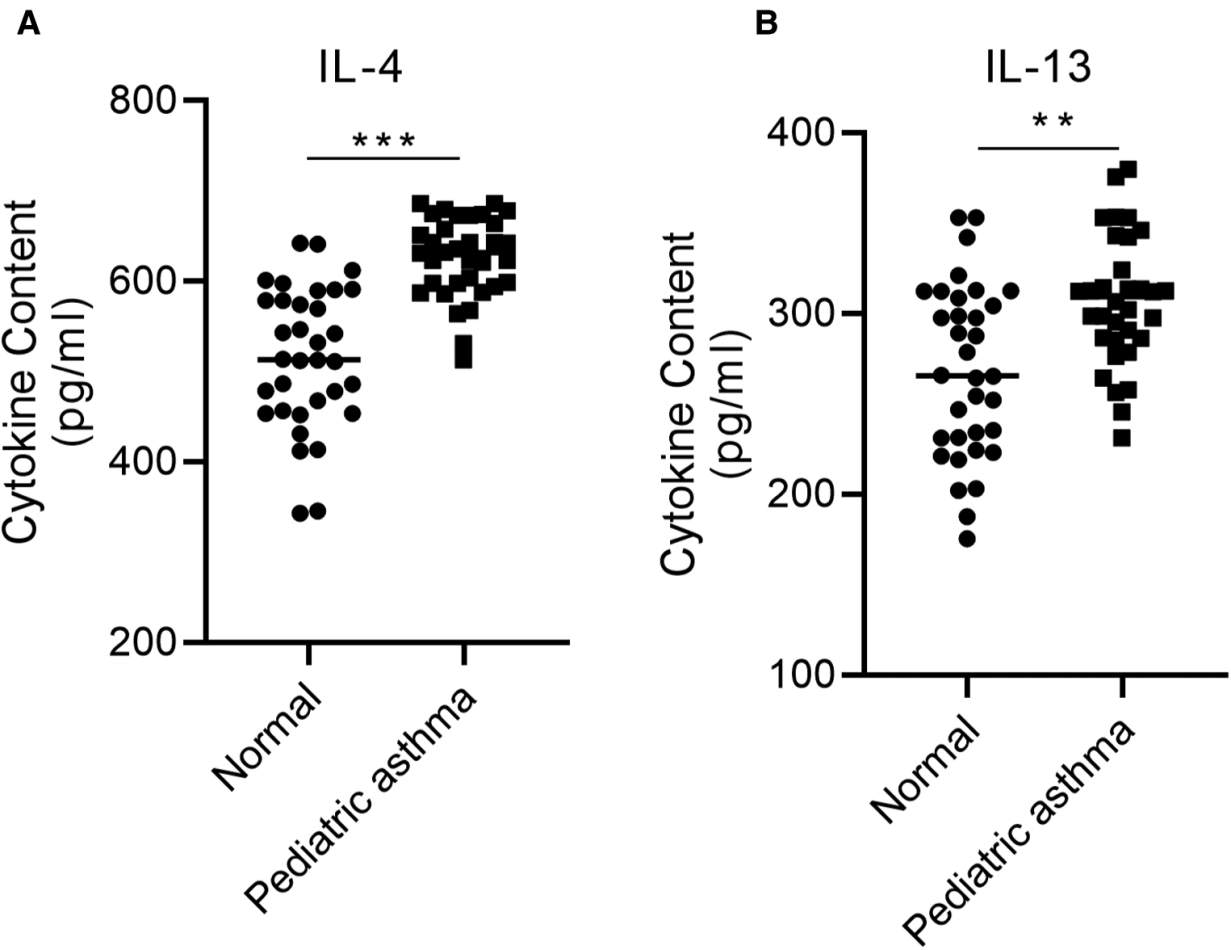
IL-4 and IL-13 are highly expressed in the serum of children with asthma. The concentration of (**A**) IL-4 and (**B**) IL-13 in the blood of asthmatic children relative to healthy controls was assessed *via* ELISA. **p < 0.01, ***p < 0.001.

Pediatric asthma is a chronic inflammatory airway disease associated with type 2 cytokines, IL-4, and IL-13 (17). Therefore, the levels of IL-4 and IL-13 were investigated in the blood of healthy children and children with asthma using ELISA. The concentration of IL-4 and IL-13 was significantly increased in the blood of pediatric asthma patients relative to the healthy control group (Figure 3A,B).

### *CLCA1* expression level is highly correlated with IL-13 content

**Figure 4:**
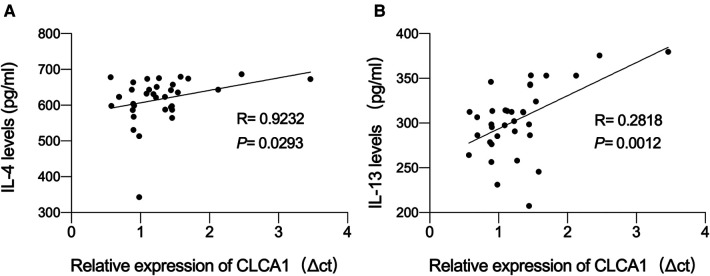
*CLCA1* expression level is highly correlated with IL-13 content. (**A,B**) Person correlation analysis of the correlation of *CLCA1* and IL-4 expression and the correlation of *CLCA1* and IL-13 expression in the blood of healthy children and asthmatic children.

Based on the ELSA data, we hypothesized that the expression level of *CLCA1* in the blood may be related to IL-4 and IL-13 secretion. To determine whether the expression level of *CLCA1* is correlated with blood IL-4 and IL-13 concentration, a correlation analysis of *CLCA1* expression and serum IL-4 and IL-13 was performed. *CLCA1* expression was weakly correlated with secreted IL-4 (Figure 4A), and highly correlated with secreted IL-13 (Figure 4B).

### IL-13 stimulation enhances *CLCA1* expression and downregulates bronchial epithelial cell function *in vitro*

**Figure 5:**
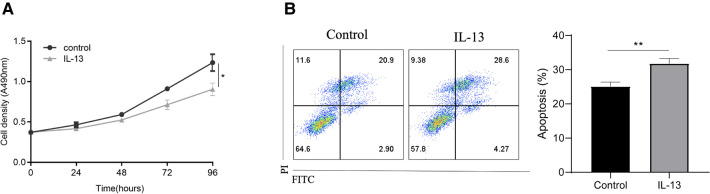
IL-13 stimulation enhances *CLCA1* expression and downregulates bronchial epithelial cell function *in vitro*. BEAS-2B cells were stimulated with IL-13. Following stimulation, (**A**) cell proliferation was assessed using the CCK-8 assay and (**B**) apoptotic cells were assessed *via* flow cytometry. *p < 0.05, **p < 0.01.

To validate the role of IL-13 in pediatric asthma, bronchial epithelial cells (BEAS-2B cells) were stimulated with IL-13 to mimic asthma *in vitro*. IL-13 stimulation reduced the viability of BEAS-2B cells (Figure 5A). Additionally, flow cytometric analysis revealed that IL-13 stimulation promoted BEAS-2B apoptosis (Figure 5B).

### Knockdown of *CLCA1* attenuates IL-13-induced bronchial epithelial cell activity and reduces cell apoptosis

**Figure 6:**
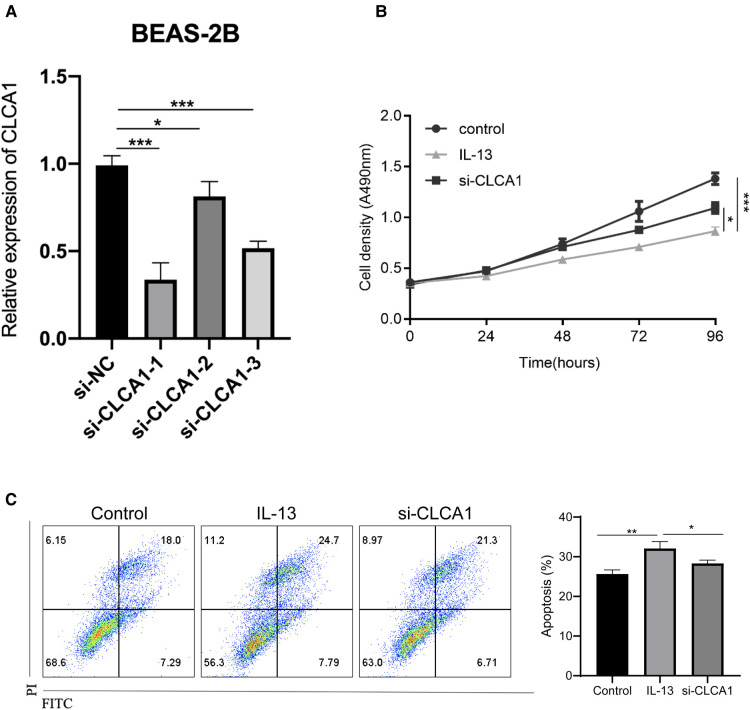
Knockdown of *CLCA1* attenuates IL-13-induced bronchial epithelial cell activity and reduces cell apoptosis. *CLCA1* knocked down BEAS-2B cells were stimulated with IL-13 and their (**A,B**) cell proliferation ability was assessed using the CCK-8 assay. (**C**) Flow cytometry was used to detect apoptotic cells.*p < 0.05,***p < 0.001.

As *in vitro* IL-13 stimulation reduced the activity of bronchial epithelial cells and promoted apoptosis, we next aimed to determine the specific effect of *CLCA1*. To achieve this, *CLCA1* was knocked down in BEAS-2B cells using siRNA. The knocked-down cells were then stimulated with IL-13 (Figure 6A). *CLCA1* knockdown rescued the IL-13-induced reduction in bronchial epithelial cell viability (Figure 6B) as well as apoptosis (Figure 6C).

## Discussion

*CLCA1* is hypothesized to be widely involved in the pathogenesis of inflammatory airway diseases such as asthma. *CLCA1* was bioinformatically identified as an aberrantly expressed gene in pediatric asthma. Here, we confirm that *CLCA1* was indeed significantly overexpressed in the blood of asthmatic children relative to healthy controls. Additionally, elevated levels of IL-4 and IL-13 were detected in the serum of children with asthma, and IL-13 was positively correlated with the expression of *CLCA1*. In vitro IL-13 stimulation significantly reduced the viability of lung endothelial cells (BEAS-2B) and promoted apoptosis. Interestingly, *CLCA1* knockdown reversed the IL-13-induced damage to BEAS-2B cells. Together, these results suggest that *CLCA1* mediates the regulatory effect of IL-13 on pediatric asthma.

Early identification and intervention are key strategies for controlling childhood asthma. However, novel strategies that allow for the prevention of childhood asthma would be even more important. At present, many studies indicate that genetic factors are an important factor contributing to pediatric asthma (18, 19). In German children, polymorphisms in variants of the homeobox transcription factor gene, HLX1, were correlated with an increased risk of childhood asthma (18). Additionally, a study of gene variants in T-cell-specific TBX21, a factor that induces TH1 differentiation and blocks TH2 commitment together with HLX1, suggests that TBX21 polymorphisms contribute to asthma, possibly through altered TBX21 promoter activity (19). Together, these studies suggest that a better understanding of the functional effects of gene-related polymorphisms could help identify key pathways in childhood asthma development.

Several previous studies suggest that respiratory infections may be associated with childhood asthma. In the National Health and Nutrition Examination Survey, Gergen et al. examined the relationship between total IgE levels and asthma. Those authors noted that total IgE levels were associated with asthma only in persons with positive results for at least 1 allergen-specific IgE (20). In addition, cytokines appear to be strongly associated with childhood asthma. Previous studies have shown that the IL-26 secretion can be used as a biomarker for childhood asthma (21). Additionally, IL-10 and IL-1β gene polymorphisms are associated with allergic asthma in children (22, 23) and the association between IL-13 polymorphisms and susceptibility to childhood asthma has been extensively studied (24–26). Consistent with these findings, our experimental results show that IL-13 has a significant correlation with pediatric asthma.

Studies have shown that *CLCA1* plays an important role in a mouse model of dextran sulfate sodium-induced colitis by the modulation of early immune responses through altered cytokine secretion (27). Furthermore, *CLCA1* deficiency resulted in decreased cytokine expression and decreased leukocyte recruitment in an acute pneumonia mouse model (28). Finally, *CLCA1* has been shown to activate macrophages *in vitro*. Consistent with our findings, previous studies have shown that, in normal human bronchial epithelial cells, IL-13 receptor activation leads to STAT6 activation and subsequent induction of *CLCA1* gene expression (29). Furthermore, we demonstrated that the expression of IL-13 is highly correlated with *CLCA1* in pediatric asthma. Together, these data indicate that *CLCA1* may play an important role in pediatric asthma.

While this study suggests that *CLCA1*-mediated IL-13 has a role in the regulation of pediatric asthma, several aspects remain to be improved. Firstly, a limited number of serological samples from asthmatics patients were assessed. In future studies, the number of included asthmatic patients that should ideally be representative of different global sociodemographic populations should be increased to ensure sufficient power to identify robust biological phenomena. Secondly, the regulatory effects of *CLCA1* and IL-13 on airway epithelial cell function in cell lines were only assessed *in vitro*. Therefore, the role of *CLCA1* and IL-13 should be robustly verified in animal models. Finally, *CLCA1* is associated with inflammatory airway disease; however, its role in pediatric asthma had not been reported. Whilst this study implicates the potential role of *CLCA1* in pediatric asthma, additional in-depth investigations into the biological mechanism are still required.

In conclusion, this study shows that the expression of *CLCA1* and IL-13 was positively correlated in pediatric asthma, and that knockdown of *CLCA1* attenuated the IL-13-induced decreased activity and apoptosis of bronchial epithelial cells. These findings provide new research insights and elucidate potential therapeutic targets for pediatric asthma.

## Data Availability

Publicly available datasets were analyzed in this study. This data can be found here: GSE103166; https://www.ncbi.nlm.nih.gov/geo/.
